# Schwere degenerative Veränderungen des Kiefergelenks in Verbindung mit diffus-sklerosierender Osteomyelitis

**DOI:** 10.1007/s00106-025-01649-6

**Published:** 2025-08-12

**Authors:** Ina Dewenter, Sven Otto, Tim Hildebrandt, Wenko Smolka, Christoph A. Reichel, Katharina Theresa Obermeier

**Affiliations:** 1 Klinik und Poliklinik für Mund‑, Kiefer- und plastische Gesichtschirurgie, Klinikum Universität München, Ludwig-Maximilians-Universität München, Lindwurmstr. 2a, 80337 München, Deutschland; 2 Klinik und Poliklinik für Hals-Nasen-Ohrenheilkunde, Ludwig-Maximilians-Universität München, München, Deutschland

**Keywords:** Kieferschmerzen, Otitis media, Mastoiditis, Temporomandibulargelenk, Arthrose, Jaw pain, Otitis media, Mastoiditis, Temporomandibular joint, Osteoarthritis

## Abstract

**Hintergrund:**

Die diffus-sklerosierende Osteomyelitis (DSO) ist eine seltene chronische Erkrankung, die mit starken Kieferschmerzen, Schwellung des Unterkiefers und Trismus einhergeht. Eine Beteiligung des Kiefergelenks ist selten, über Fälle mit schwerer Kiefergelenkzerstörung bei DSO-Patienten wurde bislang nur selten berichtet.

**Fallbericht:**

Der folgende Fallbericht beschreibt den Krankheitsverlauf einer 26-jährigen Patientin mit Zustand nach akuter linksseitiger Otitis media, chronischer Mastoiditis und Osteomyelitis der Schädelbasis, die im Verlauf eine DSO im linken Unterkiefer mit schweren degenerativen Veränderungen des Kiefergelenks entwickelte. Die Verabreichung von 6 mg Ibandronsäure in Kombination mit einer Arthrozentese und einer arthroskopischen Instillation von Hyaluronsäure in den Kiefergelenkraum führte zu einer Remission der Symptome.

**Schlussfolgerung:**

Eine Mastoiditis mit Schädelbasisosteomyelitis, mutmaßlich verursacht durch eine akute Otitis media, kann zu degenerativen Veränderungen im Kiefergelenk führen, insbesondere bei prolongiertem Verlauf oder fortschreitender entzündlicher Ausbreitung. Die Kombination mit der Entwicklung einer DSO des Unterkiefers stellt eine mögliche Komplikation dar. Das Bewusstsein für das Zusammentreffen der genannten Erkrankungen mit ausstrahlenden präaurikulären Schmerzen ist von entscheidender Bedeutung für die korrekte Diagnose und für ausreichende Behandlungsprotokolle.

## Abstract (en)

**Background:**

Diffuse sclerosing osteomyelitis (DSO) is a rare chronic disease, which is associated with severe jaw pain, swelling of the mandible and trismus. Involvement of the temporomandibular joint is rare and reports on cases with severe destruction of the temporomandibular joint in DSO patients are so far uncommon.

**Case report:**

The following case report describes a 26 year-old female patient, who presented with a acute otitis media, followed by chronic mastoiditis and osteomyelitis of the skull base in her medical history, and who developed DSO in the left mandible with severe degenerative changes of the TMJ in the course of the disease. The administration of 6mg ibandronic acid in combination with arthrocentesis and arthroscopic instillation of hyaluronic acid in temporomandibular joint space led to remission of the symptoms.

**Conclusion:**

Mastoiditis with osteomyelitis of the skull base, presumably caused by an acute otitis media, can lead to degenerative alterations of the temporomandibular joint, especially in cases of a prolonged course and progressive spread of inflammation. The combination with the development of a DSO of the mandible represents a possible complication. Awareness for the coincidence of the named diseases with radiating preauricular pain is of decisive importance for the correct diagnosis and for sufficient treatment protocols.

## Anamnese

**Abb. 1:**
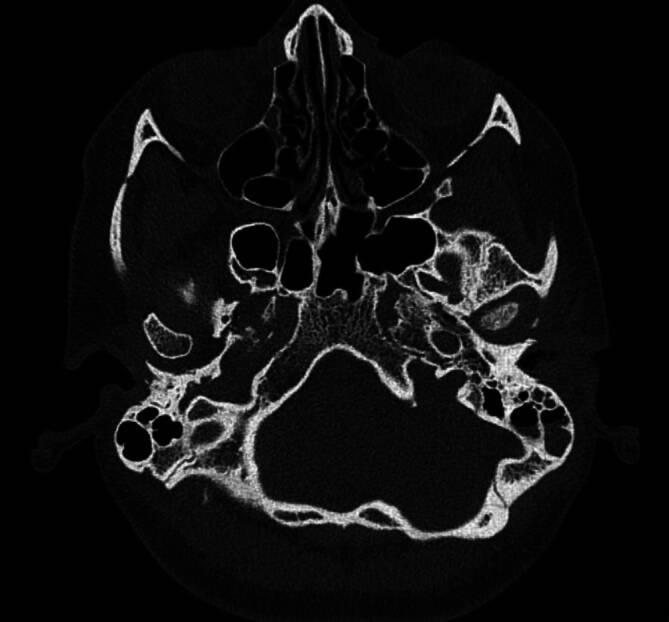
Computertomographie des Felsenbeins bei initialem Symptombeginn: vereinzelt verlegte Mastoidzellen links

**Abb. 2:**
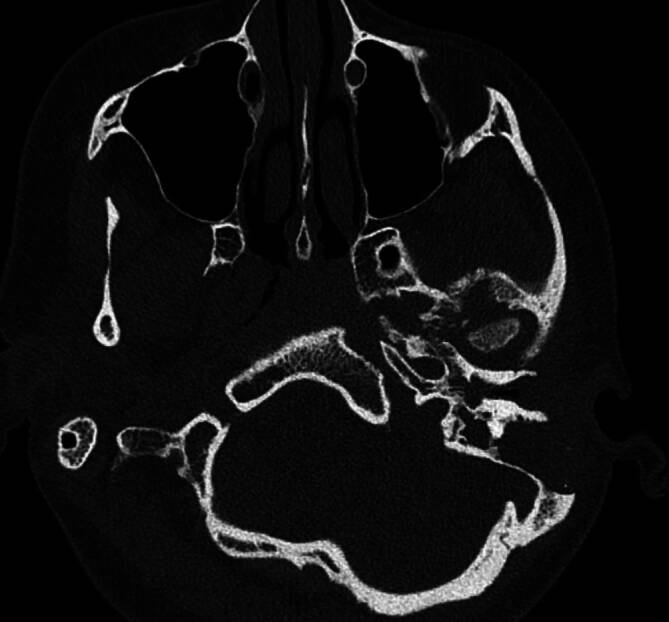
Computertomographie des Felsenbeins bei Osteomyelitis der Schädelbasis sowie der Fossa articularis und des Caput mandibulae

Eine 26-jährige Patientin stellte sich in der Klinik für Mund‑, Kiefer- und Gesichtschirurgie der Ludwig-Maximilians-Universität München mit einer akuten Schmerzexazerbation im Bereich des linken Unterkiefers vor. Sie gab an, dass die Symptome bereits seit 5 Jahren bestehen. Initial wurde die Patientin *ex domo* aufgrund einer Otitis media acuta mit toxischem Innenohrabfall, Otalgie und einer Verminderung des Hörvermögens auf der betroffenen linken Seite vorstellig (kombinierte Schwerhörigkeit mit einem Innenohrabfall von 30–40 dB und einer Schallleitungskomponente von ca. 20 dB). Zusätzlich zeigte sich ein linksseitiges Gehörgangfurunkel. Es erfolgten zunächst eine linksseitige Parazentese mit Paukendrainage sowie die Inzision des Gehörgangfurunkels. Im Verlauf der Erkrankung wurde bei rezidivierenden Schmerzen sowie schleimiger Otorrhö und radiologisch-bildmorphologisch verlegten Mastoidzellen (Abb. 1) eine linksseitige Mastoidektomie bei chronischer Mastoiditis durchgeführt. Wenige Wochen später manifestierten sich bei der Patientin eine linksseitige Fazialisparese (Augenast- sowie Mundastschwäche, House-Brackmann-Grad II) sowie starke linksseitige Kiefergelenkschmerzen. In der durchgeführten Computertomographie (CT) zeigte sich eine Osteomyelitis in den ventrobasalen Anteilen des Felsenbeins und in der Fossa mandibularis des Kiefergelenks links mit osteolytischer Destruktion des Knochens und des Kiefergelenkköpfchens (Abb. 2). Es folgten eine prolongierte Antibiotikatherapie mittels Clindamycin über 6 Wochen und eine hyperbare Sauerstofftherapie. Unter der Therapie kam es zunächst zu einer Remission der Symptomatik. Audiologisch persistierte eine Schallleitungsschwerhörigkeit im Tieftonbereich von bis zu 20 dB.

In den darauffolgenden 4 Jahren berichtete die Patientin über wiederkehrende Schmerzepisoden im Kiefergelenk, die alle 1 bis 2 Monate auftraten und etwa 3 bis 4 Wochen andauerten. Eine Schienentherapie mittels Äquillibrierungsschiene und regelmäßiger Physiotherapie im Bereich der Kiefergelenke führten zu einer leichten Regredienz der Symptomatik. Zuletzt entwickelte sich jedoch eine über Wochen andauernde akute Exazerbation der Schmerzen im linken Unterkiefer mit Gelenkbeteiligung.

## Klinische Untersuchung

**Abb. 3:**
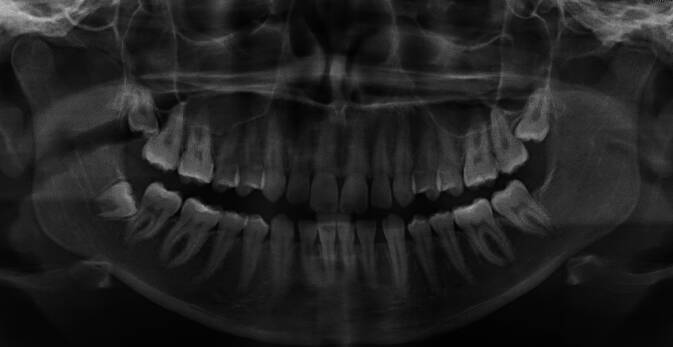
Orthopantomographie: sklerotische Bereiche im linken aufsteigenden Unterkiefer; linker Kondylus mit schweren degenerativen Veränderungen mit Verschmälerung des Gelenkspalts

**Abb. 4:**
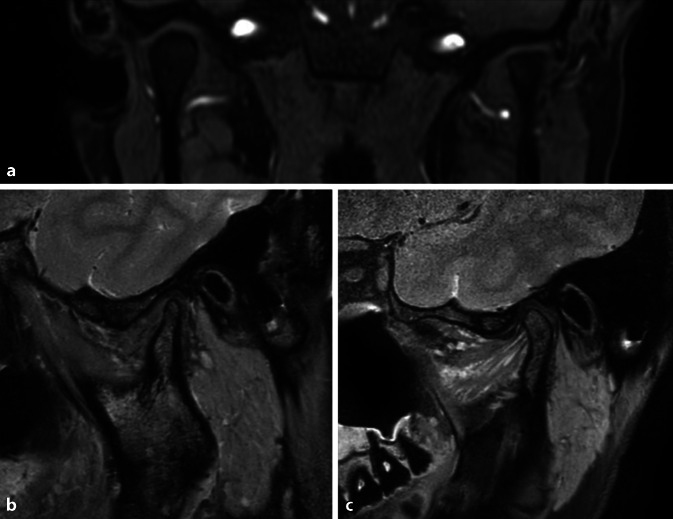
MRT(Magnetresonanztomographie)-Bildgebung des Kiefergelenks: **a** koronare Schicht mit Resorption des linken Kiefergelenkköpfchens, **b** sagittale Schicht mit Atrophie des linken Kiefergelenkköpfchens, **c** sagittale Schicht des nicht betroffenen rechten Kiefergelenks

In der klinischen Untersuchung zeigte sich eine diskrete Schwellung im Bereich des linken Unterkiefers, schmerzhaft auf Palpation. Der intraorale Befund zeigte sich ohne pathologischen Befund. In der Orthopantomographie präsentierte sich eine Sklerose im Bereich des aufsteigenden Astes des linken Unterkiefers (Abb. 3). Die Magnetresonanztomographie (MRT) der Kiefergelenke zeigte einen aufgetriebenen Ramus mandibulae auf der linken Seite mit einem inhomogenen Kontrastsignal, vereinbar mit einer Osteomyelitis (Abb. 4). Darüber hinaus zeigte sich eine Atrophie des linken Kiefergelenks. Im Seitenvergleich konnten eine Kontrastverstärkung mit Ödem im Musculus masseter und im medialen Musculus pterygoideus auf der linken Seite sowie eine diskrete Kontrastverstärkung des Nervus alveolaris inferior auf der linken Seite beobachtet werden. Die intra- und periparotidealen Lymphknoten zeigten sich auffällig vergrößert.

## Therapie

Nach klinischer und radiologischer Bestätigung der Diagnose einer diffus-sklerosierenden Osteomyelitis (DSO) wurde eine 1‑malige intravenöse Therapie mit Ibandronat 6 mg und Ibuprofen eingeleitet. Auf diese Weise wurde eine Schmerzlinderung erreicht, die zunächst 5 Wochen anhielt. Das Vorgehen wurde nach 6 Wochen aufgrund einer erneuten Schmerzexazerbation wiederholt. Die Patientin stellte sich 12 Wochen später mit einer erneuten Schmerzexazerbation im linken Kiefergelenk, einer Schwellung des linken Unterkiefers und einer eingeschränkten Mundöffnung von 26 mm vor. Aufgrund der in der MRT nachgewiesenen linksseitigen Kiefergelenkdegeneration wurde im weiteren Verlauf eine Kiefergelenkarthroskopie mit Arthrozentese und intraoperativer Instillation von Hyaluronsäure durchgeführt. Zusätzliche Physiotherapie und Schienentherapie waren Teil der postoperativen Behandlungsverfahren, die schließlich zu einer kompletten Remission der Symptome führten.

## Diskussion

Die DSO ist eine chronische Erkrankung, die hauptsächlich den Unterkiefer betrifft. Zu den klinischen Symptomen gehören Schwellung des Unterkiefers mit periostaler Reaktion sowie unregelmäßig auftretende starke Kieferschmerzen, die häufig in Episoden auftreten, gefolgt von symptomfreien Intervallen [1].

Die Kiefergelenkarthritis ist eine häufig berichtete Komplikation der Otomastoiditis, welche eine typische Komplikation der akuten Otitis media ist. Luscan et al. zeigten, dass 15 von 45 Patienten mit Otomastoiditis auch einen Kiefergelenkerguss aufwiesen. Von diesen Patienten wiesen 6 Patienten einen Abszess und 2 Patienten klinische Ankylosen auf [2]. Da unsere Patientin zunächst mit akuter Otitis media, dann mit chronischer Mastoiditis und später mit einer Schädelbasisosteomyelitis vorstellig wurde, muss in Betracht gezogen werden, dass diese Infektion mit hoher Wahrscheinlichkeit ein Auslöser für die degenerativen Veränderungen im linken Unterkieferkondylus gewesen ist [3]. Bei der Betrachtung der ätiologischen Aspekte der DSO wird angenommen, dass die Krankheit auch durch eine (orale) Infektion verursacht wird, die sich zu einer chronischen sterilen Osteomyelitis entwickelt. Darüber hinaus besteht ferner die Hypothese, dass eine erhöhte osteoblastische Aktivität aufgrund von Störungen im RANK(„receptor activator of NF-κB“)/RANKL(„RANK ligand“)/OPG(Osteoprotegerin)-System zu einer Dysregulation der Osteoklasten- und Osteoblastenaktivität führt, was wiederum die klinischen Symptome von DSO-Patienten erklären könnte [4]. Somit könnten sowohl eine orale als auch eine otogene Infektion als Auslöser für die DSO und die Kiefergelenkdegeneration fungieren.

Zudem wurde die DSO im Rahmen der Manifestation des SAPHO(„synovitis, acne, pustulosis, hyperostosis, and osteitis“)-Syndroms beschrieben [5]. Zu den definierten Diagnosekriterien gehören multifokale Osteomyelitiden mit oder ohne Hautmanifestationen, sterile akute oder chronische Gelenkentzündungen in Verbindung mit Pusteln oder Psoriasis an den Handflächen und Fußsohlen, Akne oder Hidradenitis sowie sterile Osteitis bei Vorliegen einer der Hautmanifestationen [6]. Da bei unserer Patientin sowohl eine Osteomyelitis als auch eine chronische Entzündung des Kiefergelenks mit einer Hautmanifestation im Sinne des Gehörgangfurunkels auftrat, muss diagnostisch auch die DSO im Rahmen eines SAPHO-Syndroms diskutiert werden.

Im vorliegenden Fall stellten die DSO-Manifestationen wie Schwellung und verdickte Kortikalis sowie die rezidivierende Schmerzmanifestation mit symptomfreien Intervallen einen typischen Krankheitsverlauf dar. Die initiale Behandlung, die *alio loco* durchgeführt wurde, umfasste konservativere Behandlungsansätze wie Steroid- oder Analgetikamedikation, Aufbissschienentherapie und Physiotherapie, über die in der Literatur häufig berichtet wird [7], und führte in den frühen Stadien der Erkrankung zu einer Remission der Symptome. Später erfolgte die intravenöse Ibandronatgabe (6 mg), unter der es zu einer weiteren Remission der Symptomatik kam und die bereits früh als vielversprechender Therapieansatz bei DSO-Patienten beschrieben wurde [8]. Dennoch konnte mit dieser Behandlung keine vollständige Schmerzkontrolle erreicht werden, da die Patientin neben der DSO auch eine linksseitige Kiefergelenkosteoarthritis aufwies. Die Kiefergelenkosteoarthritis („temporomandibular joint osteoarthritis“, TMJOA) wird als eine Kombination aus degenerativer Gelenkerkrankung und Gelenkschmerzen angesehen [9]. Entzündung und Umbau des subchondralen Knochens im Frühstadium der TMJOA werden als möglicher Mechanismus für die Entstehung und das Fortschreiten der TMJOA angesehen [10]. Zu den therapeutischen Ansätzen gehört die Arthrozentese, die bei Patienten, bei denen eine TMJOA diagnostiziert wurde, die Schmerzen wirksam reduziert und die Kieferfunktion verbessert. Auch die Verabreichung von Hyaluronsäure („hyaluronic acid“, HA) kann eine positive Wirkung erzielen [11]. Bei unserer Patientin führte eine Kombination aus Arthrozentese und HA-Gabe zu einer Remission der Symptome.

## Fazit für die Praxis

- Der vorliegende Fallbericht legt nahe, dass eine chronische Mastoiditis mit Schädelbasisosteomyelitis, verursacht durch eine akute Otitis media, zu degenerativen Veränderungen im Kiefergelenk führen kann, insbesondere bei chronischem Verlauf oder fortschreitender entzündlicher Ausbreitung.
- Die Kombination mit der Entwicklung einer diffus-sklerosierenden Osteomyelitis stellt eine außergewöhnliche Konstellation dar, die in der Literatur bislang nur vereinzelt beschrieben wurde.
- Daher ist das Bewusstsein für das Zusammentreffen der genannten Erkrankungen mit ausstrahlenden präaurikulären Schmerzen von entscheidender Bedeutung für die korrekte Diagnose und für ausreichende Behandlungsprotokolle.
- Durch eine enge interdisziplinäre Zusammenarbeit können frühzeitig eine gute Schmerzkontrolle und eine effektive Symptomkontrolle erreicht werden, was entscheidend für den Behandlungserfolg ist.
